# The effectiveness and safety of extracorporeal shock wave therapy (ESWT) on spasticity after upper motor neuron injury: A protocol of systematic review and meta-analysis

**DOI:** 10.1097/MD.0000000000018932

**Published:** 2020-02-07

**Authors:** Dan-Yang Liu, Dong-Ling Zhong, Juan Li, Rong-Jiang Jin

**Affiliations:** School of Health Preservation and Rehabilitation, Chengdu University of Traditional Chinese Medicine, Chengdu, Sichuan, China.

**Keywords:** spasticity, extracorporeal shock wave therapy, upper motor neuron injury, randomized controlled trial, meta-analysis, systematic review

## Abstract

**Background:**

Spasticity is one of the manifestations of motor dysfunction in upper motor neuron syndrome, which is characterized by increased muscle tone. Spasticity seriously affects the motor function and activity of daily life of patients. Some studies have shown that extracorporeal shock wave therapy (ESWT) can relieve spasticity in recent years. However, the effectiveness and safety of ESWT on spasticity after motor neuron injury have not been confirmed. The purpose of this systematic review (SR) is to evaluate the effectiveness and safety of ESWT on spasticity after upper motor neuron injury.

**Methods:**

We will search China National Knowledge Infrastructure (CNKI), the Chinese Science and Technology Periodical Database (VIP), Wan Fang Data, China Biology Medicine (CBM), PubMed, Embase, The Cochrane Library, and Web of Science systematically from their inception dates through October 2019 to obtain randomized controlled trials (RCTs) using ESWT to relieve spasticity in patients after upper motor neuron injury. The primary outcome will be the Modified Ashworth Scale (MAS). Secondary outcomes will include Composite Spasticity Scale (CSS), Spasm Frequency Scale, Modified Tardieu Scale (MTS), electrophysiological study (ratio of maximum H reflex to maximum M response, root mean square value, integrated electromyogram, co-contraction ratio, etc.), or other spasticity-related outcomes. In addition, adverse events will also be assessed as safety measurement. Study selection, data extraction, and quality assessment will be performed independently by 2 reviewers. Assessment of risk of bias and data synthesis will be performed using Review Manager software (RevMan, version 5.3.5) and R (version 3.6.1) software.

**Results:**

We will synthesize current studies to evaluate the effectiveness and safety of ESWT on spasticity after upper motor neuron injury.

**Conclusion:**

Our study will provide evidence of ESWT on spasticity after upper motor neuron injury.

**Ethics and dissemination:**

The ethical approval is not required since SR is based on published studies. The results of this SR will be published in a peer-reviewed scientific journal according to the Preferred Reporting Item for Systematic Review and Meta-analysis (PRISMA) guidelines.

**PROSPERO registration number:**

CRD42019131059

## Abstract 2

Supplemental Digital Content is available in the text

## 1 Background

Spasticity refers to abnormal increase in muscle tone caused by upper motor neuron injury, which is related to the reduction or interruption of upper motor neuron inhibition of the descending motor pathway of the spinal cord and the overexcitation of alpha motor neuron.^[1]^ Spasticity is a common symptom in stroke, spinal cord injury, cerebral palsy, and multiple sclerosis.^[2,3]^ When suffering from severe or long-term spasticity, patients will experience muscle fibrosis, pain, joint stiffness or even contracture,^[4]^ which seriously affects patients’ motor function and activity of activities.^[5]^ Spasticity also brings inconvenience to family care, and hinders the patient from returning to the family, study, work and society.^[6]^

The methods of relieving spasticity include passive stretching,^[7]^ physical therapy,^[8]^ extremity casting,^[9]^ anti-spasticity drugs,^[10]^ botulinum toxin A (BTX-A) injection,^[11]^ and surgery.^[12]^ However, spasticity is difficult to be treated due to unwanted side effects or inadequacy of the methods mentioned above.^[13]^ For example, the effectiveness and long-term effectiveness of physical therapy need further evaluation^[14]^; anti-spasticity drugs may reduce the power of normal muscles, and their effects may decrease over time.^[15]^ In addition, repetitive injections of BTX-A may stimulate the formation of neutralizing antibodies and produce the state of “no response”.^[16]^ Therefore, it is necessary to find an effective and safe therapy to relieve spasticity and promote recovery for patients with spasticity. Currently, extracorporeal shock wave therapy (ESWT) is considered as a new therapeutic method for spasticity. Clinical studies have found that ESWT can relieve spasticity caused by cerebral palsy,^[17]^ stroke^[18]^ and multiple sclerosis.^[3]^

There are 5 previous systematic reviews (SRs)^[2,15,19–21]^ about ESWT on spasticity after brain injury. We assessed the methodological quality of them with the A Measurement Tool to Assess systematic Reviews 2.0 (AMSTAR 2.0).^[22]^ All of the 5 SRs showed positive results, however, the methodological quality of 4 SRs^[2,15,19,21]^ were critically low and 1 SR^[20]^ was moderate. Only 1 SR did a pre-defined protocol and searched grey literature. All SRs ignored funding sources of included trails and did not provide a list of excluded studies (Table 1). For the above reasons, we plan to conduct an SR and meta-analysis to evaluate the effectiveness and safety of ESWT on spasticity after upper motor neuron injury according to AMSTAR 2.0 and report in compliance with the Preferred Reporting Items for Systematic Reviews and Meta-Analyses (PRISMA).^[22,23]^

**Table 1:**
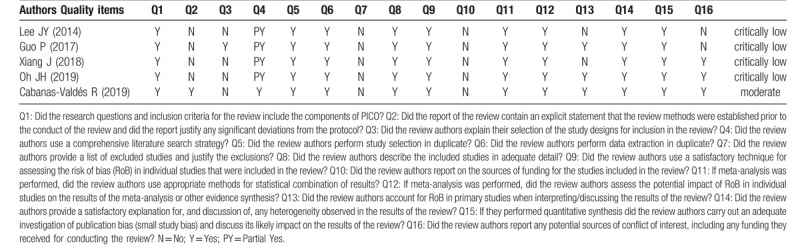
Methodological quality by AMSTAR 2.0. AMSTAR 2.0 = A Measurement Tool to Assess systematic Reviews 2.0.

## 2 Methods

### 2.1 Study registration

The protocol of this SR has been registered in PROSPERO (http://www.crd.york.ac.uk/PROSPERO). The registration number is CRD42019131059. The further amendments and rationales will be tracked in the PROSPERO. The protocol was reported according to the Preferred Reporting Items for Systematic Review and Meta-analysis Protocols (PRISMA-P) statement guidelines.

### 2.2 Inclusion criteria

#### 2.2.1 Type of studies

Only randomized controlled trials (RCTs) of ESWT on spasticity after upper motor neuron injury published in Chinese or English will be included. There will be no restriction on publication date.

#### 2.2.2 Type of participants

Participants with spasticity after upper motor neuron injury (stroke, cerebral palsy, multiple sclerosis, Parkinson's disease, etc.) will be included. There will be no restrictions on age, gender, race or nation.

#### 2.2.3 Type of interventions

RCTs used ESWT as a sole treatment or in combination with usual care or routine rehabilitation training (physiotherapy, occupational therapy, orthotics, etc.) to treat spasticity after upper motor neuron injury will be included. There will be no limit on the parameters of ESWT.

#### 2.2.4 Type of comparators

The comparative interventions will be usual care, routine rehabilitation training (physiotherapy, occupational therapy, orthotics, etc.) or sham stimulation.

#### 2.2.5 Outcome measurements

The primary outcome will be the Modified Ashworth Scale (MAS). Secondary outcomes will include Composite Spasticity Scale (CSS), Spasm Frequency Scale, Modified Tardieu Scale (MTS), electrophysiological study (ratio of maximum H reflex to maximum M response, root mean square value, integrated electromyogram, co-contraction ratio, etc.), or other spasticity-related outcomes. Moreover, adverse events (pain, petechiae, numbness, etc.) will also be assessed as safety measurement.

### 2.3 Exclusion criteria

The exclusion criteria include:

1. Cross-over RCTs, conference papers, reviews, case-control studies, array studies, case reports;
2. ESWT combined with other treatments (except usual care and routine rehabilitation);
3. Duplicate publications or the data cannot be extracted;
4. Full text cannot be obtained through various approaches;
5. RCTs in which relevant outcome indexes were not reported.

### 2.4 Search strategy

We will search China National Knowledge Infrastructure (CNKI), the Chinese Science and Technology Periodical Database (VIP), Wan Fang Data, China Biology Medicine (CBM), PubMed, Embase, The Cochrane Library, and Web of Science systematically from their inception dates through October 2019 to obtain RCTs using ESWT to relieve spasticity in patients after upper motor neuron injury. The following key search terms will be performed: extracorporeal shock wave therapy, upper motor neuron injury, spasticity and randomized controlled trial. The search strategy of PubMed is listed in Supplemental Digital Content (Appendix 1). In addition, the search strategy will be tailored according to the characteristic of the databases mentioned above.

### 2.5 Studies selection

**Figure 1:**
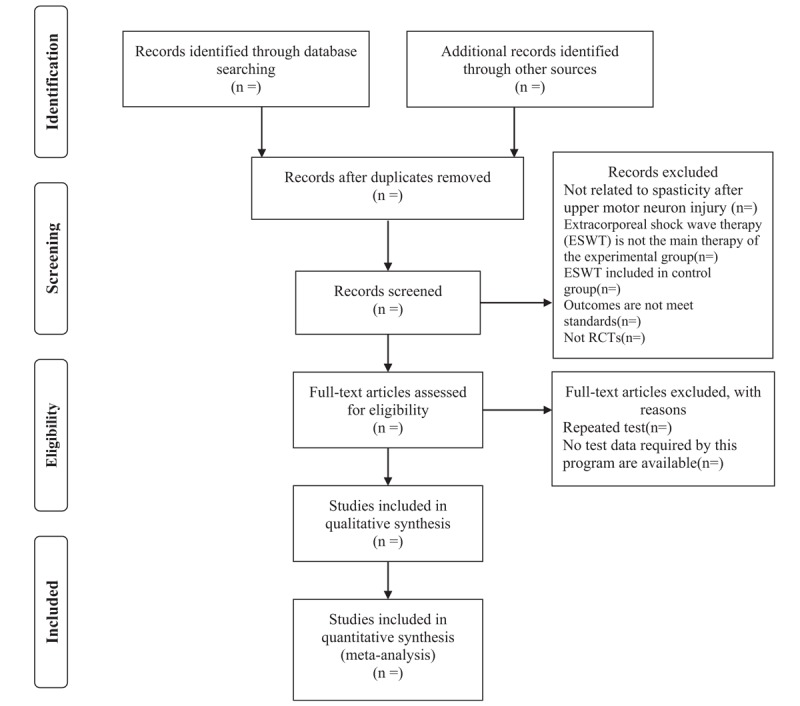
PRISMA flowchart. PRISMA = Preferred Reporting Items for Systematic Reviews and Meta-Analyses.

All the literature search results will be managed by Endnote X8, and we will identify multiple reports of the same study by matching the order of authors names, year and title. To avoid double counting we will include the most embracing report of the particular study only. Identification of eligible studies will be independently performed by 2 reviewers. Selection will be based on titles and abstracts after removing duplicates. Then, the full text will be thoroughly checked to confirm the selection criteria. Any disagreement between the 2 reviewers will be resolved by consensus, and the reasons for each excluded study will be recorded. We will check reference lists of all included studies and any relevant SRs identified for additional references. The flow chart of study selection based on PRISMA is displayed in Figure 1.

### 2.6 Data extraction

2 reviewers will extract the following data independently with a pre-defined data extraction form:

(1) study characteristics (first author, publication year and country);
(2) participant characteristics (sample size, gender, mean age, time since diseases, type of upper motor neuron injury, the degree of spasticity);
(3) methodological characteristics;
(4) results (main conclusions, adverse events, duration of follow up);
(5) key elements of risk assessment of bias
(6) sources of funding.

Authors of studies will be contacted to obtain missing data. Then the 2 reviewers cross check to ensure no mistakes. Discrepancy will be resolved through team discussion.

### 2.7 Assessment of risk of bias

The Cochrane risk of bias tool (www.cochrane-handbook.org.) will be used to assess the risk of bias independently by 2 reviewers authors. We will assess the risk of bias according to the following domains:

- random sequence generation
- allocation concealment
- blind subjects and therapists
- blind assessors
- incomplete outcome data
- selective outcome reporting
- other bias

We will grade each potential source of bias as low (meet all criteria), unclear (trials with insufficient information to judge), or high risk (meet none of the criteria). In case of doubt or disagreement, consensus will be reached through group discussions or with the participation of a third reviewer.

### 2.8 Data analysis

All statistical analyses will be conducted using Review Manager software (RevMan, version5.3.5) and R (version 3.6.1) software. The relative risk (RR) will be used to analyze dichotomous outcomes. When outcomes were continuous and in the same units, mean difference (MD) will be used. Otherwise, we will use the standardized mean difference (SMD). We will present results as a proportional effect size with confidence intervals reported based on a 95% criterion. We will define *P* ≤ .05 as statistically significant between studies. Statistical heterogeneity will be assessed by both a Cochran's Chi squared test (Q test) and an I-squared test. Fixed-effect model (FEM) will be used if acceptable heterogeneity (I^2^ ≤ 50%, *P* ≥ .1) is found. Since random-effect model (REM) is a more appropriate computational approach under conditions of heterogeneity given they are less likely to reject the null hypothesis and is more robust to large variations in sample sizes, we will use REM where significant statistical heterogeneity (I^2^ > 50%, *P* < .1) exists. Results will be described qualitatively in the text when meta-analysis is not possible.

#### 2.8.1 Dealing with missing data

We will contact the original authors via email or telephone for more information if the data is missing or unclear. If there were no reply from the original authors, we will only select and analyze the available date. Finally, we will discuss the potential impact of those missing data in the text.

#### 2.8.2 Subgroup analysis

Subgroup analysis will be carried out to investigate potential heterogeneity, based on different types of upper motor neuron injury (stroke, cerebral palsy, multiple sclerosis, Parkinson's disease, etc.), different protocols of ESWT (frequency, duration, application site, etc.), different types of comparative treatment.

#### 2.8.3 Sensitivity analysis

We will take sensitivity analysis to verify the robustness and reliability of pooled results if the data are sufficient. By excluding certain high risk of bias of studies according to Cochrane handbook or non-blinded studies, the effects of excluded studies on total pooled results will be examined.

#### 2.8.4 Publication bias

We will plan to visually inspect funnel plots to explore the likelihood of publication bias if there are at least 10 RCTs in a meta-analysis. Besides, Begg's test and Egger's test will be carried out.

### 2.9 Grading of recommendations assessment, development, and evaluation (GRADE)

GRADE system will be used by 2 independent reviewers to assess the quality of evidence of outcomes. Each outcome will be evaluated from the following 5 aspects: limitations, inconsistency, indirectness, imprecision, and publication bias.^[24]^ The quality of evidence will be graded as “high” “moderate” “low” or “very low” in accordance with the GRADE rating standards.^[25]^ The results of GRADE including evidence profile and summary of finding table will be generated using the GRADE pro software.

### 2.10 Ethics and dissemination

Ethical approval is not required for this protocol of SR since this protocol was based on published studies. The results of this SR will be published in a peer-reviewed scientific journal according to the PRISMA guidelines.

## 3 Discussion

ESWT is a new therapeutic method for spasticity.^[26]^ Latest studies suggested that ESWT appeared to involve a series of interaction between physical shock wave energy and biologic responses,^[27–29]^ including the following aspects:

1. Shock waves can stimulate the synthesis of nitric oxide which involved in formation of neuromuscular junction and physiological functions of the central nervous system^[30]^;
2. Shock waves can reduce the muscle tension and release adhesion tissues through improving microcirculation of body tissues and dissociation of high-density tissues^[31]^;
3. Promoting the release of prostaglandin-2 and P tissues, shock waves are able to expand the small and medium-sized arteries and restore blood circulation, and thus new vessels in the tissues of the active parts will be formed,^[32]^ which is critical to relieve spasticity after upper motor neuron injury.

Recent reviews have evaluated the effectiveness and safety of ESWT on spasticity after upper motor neuron injury, however, there are methodological deficiencies. Therefore, we need to perform an SR and meta-analysis by including high-quality and up-to-date studies to evaluate the effectiveness and safety of ESWT on spasticity after upper motor neuron injury. It is hoped that the results of this SR may help to establish a better method for the treatment for spasticity and provide reliable evidence for its wide application.

### 3.1 Strengths and limitations

The purpose of this SR is to evaluate the effectiveness and safety of ESWT on spasticity after upper motor neuron injury. It will be based on comprehensive searching strategies and selection of studies; data extraction and assessment of risk of bias will be conducted independently by 2 reviewers to ensure the integrity and authenticity of all relevant studies. We will assess the quality of evidence for the main outcomes following the guidelines of GRADE. The protocol of this SR has been registered in PROSPERO. However, this SR will include RCTs published in English and Chinese, a language bias may exist.

## Author contributions

Danyang Liu and Dongling Zhong contributed equally to this work. The protocol was drafted by Danyang Liu, and revised by Dongling Zhong. Methodology was developed by Juan Li. Rongjiang Jin developed the search strategy. Danyang Liu and Rongjiang Jin will independently select studies. Dongling Zhong and Juan Li will assess the risk of bias of included studies. Danyang Liu and Dongling Zhong will independently work on data extraction and synthesis.

## Supplementary Material

Supplemental Digital Content
