# Supplementary material for: The effectiveness and safety of extracorporeal shock wave therapy (ESWT) on spasticity after upper motor neuron injury: A protocol of systematic review and meta-analysis
Source: Medicine (Baltimore). 2020 Feb 7;99(6):e18932. doi: 10.1097/MD.0000000000018932 (PMC7015647; doi:10.1097/MD.0000000000018932)
Supplement: Supplemental Digital Content [file medi-99-e18932-s001.docx]

**Appendix 1.** **Search strategy for Pubmed**

| Number | Search terms |
| --- | --- |
| #1 | Extracorporeal Shockwave Therapy[Mesh] |
| #2 | “Extracorporeal Shockwave Therapy”[Title/Abstract] OR “Shock Wave Therapy”[Title/Abstract] OR “Extracorporeal Shock Wave Therapy”[Title/Abstract] OR “Shock Wave”[Title/Abstract] OR ESWT[Title/Abstract] |
| #3 | #1 OR #2 |
| #4 | Muscle Hypertonia[Mesh] |
| #5 | muscle hypertonia[Title/Abstract] OR muscle spasticity[Title/Abstract] OR muscle rigidity[Title/Abstract] OR muscle tonus[Title/Abstract] OR muscle cramp[Title/Abstract] OR dystonia[Title/Abstract] OR spasm*[Title/Abstract] OR spastic*[Title/Abstract] |
| #6 | #4 OR #5 |
| #7 | Stroke[Mesh] OR Cerebral Palsy[Mesh] OR Multiple Sclerosis[Mesh] OR Spinal Cord Injuries[Mesh] OR Parkinson Disease[Mesh] |
| #8 | stroke[Title/Abstract] OR “cerebrovascular accident”[Title/Abstract] OR “cerebral palsy”[Title/Abstract] OR CP[Title/Abstract] OR “Multiple Sclerosis”[Title/Abstract] OR MS[Title/Abstract] OR “Spinal Cord Injuries”[Title/Abstract] OR SCI[Title/Abstract] OR “Parkinson Disease” [Title/Abstract] OR “Idiopathic Parkinson's Disease”[Title/Abstract] OR upper motor neuron injury[Title/Abstract] |
| #9 | #7 OR #8 |
| #10 | randomized controlled trial[Publication Type] |
| #11 | “randomized controlled trial”[Title/Abstract] OR random*[Title/Abstract] OR placebo[Tittle/Abstract] OR RCT[Title/Abstract] |
| #12 | #10 OR #11 |
| #13 | #3 AND #6 AND #9 AND #12 |
